# Reassessing the Role of Tissue Factor Pathway Inhibitor 2 in Neoplastic and Non-Neoplastic Lesions

**DOI:** 10.3390/cancers17091447

**Published:** 2025-04-25

**Authors:** Hiroshi Kobayashi, Hiroshi Shigetomi, Shogo Imanaka

**Affiliations:** 1 Department of Gynecology and Reproductive Medicine, Ms. Clinic MayOne, 871-1 Shijo-cho, Kashihara 634-0813, Japan; shogo_0723@naramed-u.ac.jp; 2 Department of Obstetrics and Gynecology, Nara Medical University, 840 Shijo-cho, Kashihara 634-8522, Japan; hshige35@gmail.com; 3 Department of Gynecology and Reproductive Medicine, Aska Ladies Clinic, 3-3-17 Kitatomigaoka-cho, Nara 634-0001, Japan

**Keywords:** clear cell carcinoma, ovarian cancer, tissue factor pathway inhibitor 2, tumor promoter, tumor suppressor

## Abstract

Objectives: Tissue factor pathway inhibitor 2 (TFPI2) is a serine protease inhibitor that suppresses tumors by preventing extracellular matrix degradation and invasion. In many malignancies, the TFPI2 promoter hypermethylation silences its transcription, increasing tumor aggressiveness. However, TFPI2 paradoxically facilitates tumor progression in certain malignancies. Elevated circulating TFPI2 levels correlate with increased cancer aggressiveness and poor prognosis in ovarian, endometrial, and renal cell carcinoma, though the mechanisms underlying its tumor-promoting effects remain unclear. This review consolidates recent findings on TFPI2 regulation, its downstream targets in cellular homeostasis, and its prognostic significance. Additionally, we reassess TFPI2′s role in tumorigenesis, particularly in clear cell carcinoma, as well as in chronic inflammation. Methods: A comprehensive literature search was performed in PubMed and Google Scholar without time restriction. Results: TFPI2 expression is tightly regulated by transcription factors, signaling molecules, growth factors, cytokines, and epigenetic modification. TFPI2 regulates cell proliferation, inflammation, and extracellular matrix (ECM) remodeling, preserving tissue homeostasis. TFPI2 also regulates vascular endothelial and smooth muscle cell proliferation, key elements of the tumor microenvironment (TME). In the nucleus, it may modulate transcription factors to influence tumor-associated macrophage (TAM) polarization, facilitating cancer invasion. Its expression may be shaped by interactions between cancer cells and TAM activation. Beyond tumorigenesis, TFPI2 contributes to both inflammatory progression and resolution in diabetes, atherosclerosis, and preeclampsia. Conclusions: TFPI2 may interact with TAMs and inflammatory cells to regulate cell proliferation and inflammation, maintaining tissue homeostasis.

## Simple Summary

Tissue factor pathway inhibitor 2 (TFPI2) has emerged as a novel serum biomarker for ovarian cancer, gaining insurance coverage in Japan in 2021. Initially characterized as a tumor suppressor gene, TFPI2 exhibits elevated serum levels in patients with ovarian and endometrial cancers compared to healthy individuals, correlating with poor prognostic outcomes. Conventionally, tumor suppressor genes are expected to be downregulated in malignancies; however, clinical observations reveal a paradoxical increase. This review article investigates the role of TFPI2 in both non-malignant and malignant tissues to elucidate the underlying mechanisms of this discrepancy. We aim to raise global awareness among researchers and clinicians regarding the intriguing functions of TFPI2 and to promote its broader clinical application.

## 1. Introduction

Tissue factor pathway inhibitor 2 (TFPI2) is a 32 kDa Kunitz-type serine protease inhibitor that is highly expressed in the placenta [1]. As a structural homolog of TFPI1, TFPI2 shares significant similarities, particularly in the presence of the Kunitz-type inhibitory domain [1]. However, despite their structural resemblance, TFPI1 and TFPI2 are encoded by distinct genes and fulfill divergent physiological roles [1,2,3]. TFPI1 is predominantly synthesized by vascular endothelial cells, with additional production by megakaryocytes, monocytes, and smooth muscle cells [4]. Functionally, TFPI1 regulates the extrinsic coagulation cascade by inhibiting the tissue factor (TF)/factor VIIa complex and factor Xa, thereby mitigating excessive thrombin generation [3,5]. In contrast, TFPI2 exhibits a comparatively weaker inhibitory effect on TF-initiated thrombin generation but demonstrates potent inhibitory activity against plasmin, the principal enzyme in fibrinolysis responsible for thrombolysis [6]. Consequently, TFPI2 is more prominently involved in extracellular matrix remodeling and angiogenesis than in direct hemostatic regulation [2,7,8,9,10]. By inhibiting plasmin, TFPI2 may attenuate fibrin degradation and clot resolution, thereby elevating the risk of thrombosis [3]. Additionally, TFPI2 localized in platelet α-granules may further contribute to thrombolysis inhibition [6]. Thus, while both TFPI1 and TFPI2 participate in coagulation regulation, they target distinct components of the hemostatic system and assume unique roles in pathophysiological processes. Notably, TFPI2 has emerged as a promising biomarker for predicting asymptomatic venous thromboembolism (VTE) in ovarian cancer patients [3,11,12].

Furthermore, numerous studies have reported that TFPI2 downregulation in cancer cells facilitates tumor progression by promoting extracellular matrix degradation and remodeling, underscoring its role as a tumor suppressor gene [13,14]. Recently, two compelling review articles have provided valuable insights into the multifaceted functions of TFPI2 in oncogenesis. Wojtukiewicz et al. [15] presented a comprehensive update on the impact of TFPI2 on tumor progression and metastasis, while Kobayashi et al. [16] explored the association between elevated serum TFPI2 levels and prognosis in ovarian and endometrial cancers, proposing that TFPI2 may paradoxically function as a tumor-promoting factor in these malignancies. With the establishment of robust TFPI2 quantification assays, it has become evident that circulating concentrations of TFPI2 are markedly elevated in ovarian cancer, as well as in endometrial and renal cell carcinomas [17,18,19]. In Japan, following a multicenter prospective clinical trial, TFPI2 has been reimbursed by health insurance as a serum-based diagnostic biomarker for ovarian cancer since 2021 [20]. Moreover, a series of studies have demonstrated that increased circulating TFPI2 levels correlate with adverse prognosis in ovarian and endometrial cancers [18,21,22]. This observation presents a paradox: while TFPI2 downregulation in tumor cells is linked to cancer progression and poor prognosis [15], elevated systemic TFPI2 concentrations also appear to be associated with unfavorable clinical outcomes in select cancers.

Moreover, TFPI2 has been reported to be expressed by vascular endothelial cells, trophoblasts, inflammatory cells, and immune cells, where it exerts multifaceted effects on both the pathological progression and resolution of inflammation-associated diseases such as diabetes, arteriosclerosis, and preeclampsia [15,23,24]. To elucidate the role of TFPI2 in tumorigenesis, it is imperative to first gain a comprehensive understanding of its physiological functions in non-malignant cells. Several critical questions remain unresolved, including the precise physiological and pathological role of TFPI2 in both malignant and non-malignant conditions, the identity of non-cancerous cells that modulate TFPI2 expression, the underlying regulatory mechanisms, and the exact influence of TFPI2 on cancer prognosis. Despite the lack of a definitive consensus regarding the physiological function of TFPI2, its pathophysiological significance has garnered increasing attention due to the substantial differences in blood TFPI2 concentrations between cancer patients and healthy individuals [14]. In this review, we systematically examine the molecular regulators of TFPI2 expression, as well as the downstream targets influenced by TFPI2, summarize emerging data on its dual roles in tumor suppression and promotion, and discuss its physiological function alongside future research directions in cancer biology.

## 2. Materials and Methods

### Search Strategy and Selection Criteria

This review presents an in-depth narrative synthesis of the current literature on both fundamental and clinical aspects of TFPI2 in the context of neoplastic and non-neoplastic lesions. A systematic search of publications up to 30 November 2024 was performed using electronic databases, including PubMed (https://pubmed.ncbi.nlm.nih.gov/) and Google Scholar (https://scholar.google.com/). The search strategy employed a combination of keywords—”TFPI2”, “ovarian cancer”, “clear cell carcinoma”, “tumor suppression”, and “tumor promotion”—with Boolean operators, as detailed in Table 1, to refine and optimize the search queries. Inclusion criteria encompassed original research articles published in English, as well as pertinent studies cited in review articles. Exclusion criteria involved duplicate records, publications unrelated to the research focus, and articles published in languages other than English. During the initial screening phase, records identified through database searches underwent deduplication, followed by the exclusion of irrelevant studies based on title and abstract reviews. In the final selection stage, full-text articles were rigorously assessed to exclude studies focusing solely on TFPI1, those lacking comprehensive basic or clinical data, and studies without pathophysiological significance.

**Figure 1:**
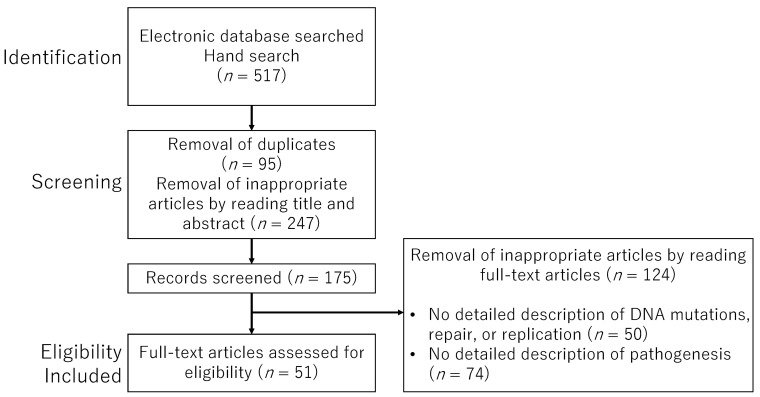
The flowchart outlines the study selection process.

The selected articles were independently reviewed by the authors (I.S. and H.S.) through a meticulous full-text evaluation process. Any disagreements or ambiguities regarding study selection were resolved through discussion and consensus. A comprehensive flowchart outlining the study selection process, including detailed inclusion and exclusion criteria, is provided in Figure 1.

An analysis of TFPI2-related publications in PubMed reveals a steady rise in studies since its identification in 1994, with a peak around 2013 followed by a subsequent decline. However, in Japan, the approval of insurance coverage for TFPI2 in April 2021 as a diagnostic aid for ovarian cancer—particularly ovarian clear cell carcinoma—has reignited research interest, leading to renewed investigations into its diagnostic and prognostic potential across a range of malignancies.

## 3. Results

### 3.1. Localization and Function of the TFPI2 Molecule

The contents of this subsection have been extensively reviewed in recent literature [15,16]; however, a concise summary is provided here to elucidate the functional role of TFPI2 in normal cells. TFPI2 is ubiquitously expressed across a wide range of human tissues, including skeletal and smooth muscle, breast, liver, pancreas, kidney, colon, stomach, esophagus, ovary, endometrium, brain, and placenta [15,25]. Expression profiles of TFPI2 mRNA in human fetal tissues are available at NCBI Gene Database, https://www.ncbi.nlm.nih.gov/gene/7980 (accessed on 30 November 2024).

Initially identified as a placental protein, TFPI2 exhibits robust expression in cytotrophoblast and syncytiotrophoblast cells of the mature placenta [26]. Beyond placental tissues, TFPI2 is physiologically synthesized in vascular endothelial cells [27], fibroblasts, platelets, macrophages, granulosa and theca cells [28], and preovulatory follicular fluid [15,29]. In vascular endothelial cells, TFPI2 is primarily localized within the extracellular matrix, although it can also be detected in the cytoplasm and nucleus [17,29,30]. In healthy tissues, TFPI2 is predominantly expressed in endothelial cells, whereas in atherosclerotic lesions, it is additionally detected in macrophages, T cells, and smooth muscle cells [27]. Functionally, TFPI2 serves as a potent inhibitor of various serine proteases, including plasmin, and modulates the activity of plasmin- and kallikrein-mediated matrix metalloproteinases (e.g., matrix metalloproteinase (MMP)-1, MMP-2, MMP-9, and MMP-13), as well as pro-MMP-1 and pro-MMP-3 [31]. By regulating extracellular matrix degradation, TFPI2 contributes to extracellular matrix stabilization, inhibits pathological angiogenesis, suppresses cellular invasion, and restrains tumor growth and metastasis [15]. Furthermore, TFPI2 plays a crucial role in apoptosis induction through both Bax- and caspase-mediated pathways by regulating the expression of death receptors and their ligands, including tumor necrosis factor alpha (TNF-α), Fas ligand (FasL), and TNFRSF1A associated via death domain (TRADD) [32,33]. Consequently, TFPI2 is widely regarded as a tumor suppressor. Beyond its involvement in cancer, aberrant expression of TFPI2 has been observed in a range of pathological conditions, including diabetes [10,24,34], atherosclerosis [35,36,37], and pregnancy-induced hypertension [23,38,39,40,41,42].

### 3.2. Molecules and Signaling Pathways Regulated by TFPI2

Beyond its role as a protease inhibitor, TFPI2 is actively involved in the modulation of intracellular signaling pathways. This subsection mainly highlights the physiological and pathological implications of TFPI2 in oncological diseases. For a discussion of TFPI2’s role in non-oncological diseases, refer to Section 3.6.

#### 3.2.1. MMP-2 Regulation by TFPI2

**Figure 2:**
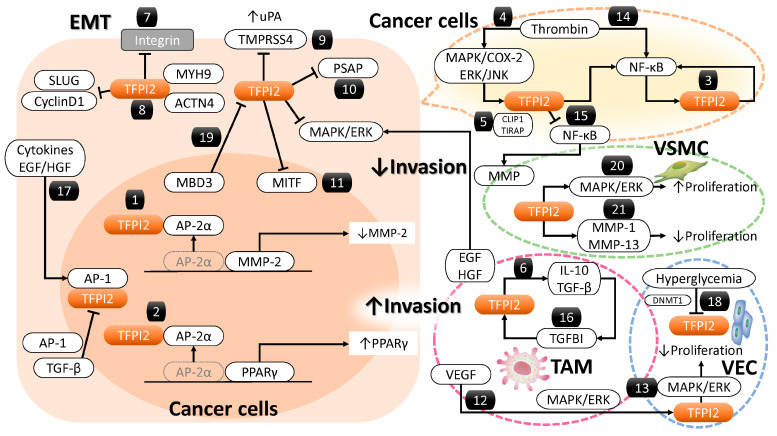
Relationship between the regulation of TFPI2 expression in cancer cells and host cells. The signaling pathway constituents numbered 1 to 21 in the figure are described in sections of the text labeled as Figure 2(1,2), and so forth. Arrows denote activation, while T-shaped symbols represent inhibition. ACTN4, actinin 4; AP-1, activator protein 1; AP-2α, activator protein 2 alpha; CLIP1, CAP-Gly domain-containing linker protein 1 (CLIP1); COX-2, cyclooxygenase-2; DNMT1, DNA methyltransferase 1; EGF, epidermal growth factor; ERK, extracellular signal-regulated kinase; HGF, hepatocyte growth factor; IL-10, interleukin-10; JNK, c-Jun N-terminal kinase; MAPK, mitogen-activated protein kinase; MBD3, methyl-CpG-binding domain protein 3; MMP, matrix metalloproteinase; MYH9, myosin 9; MITF, melanocyte-induced transcription factor; NF-κB, nuclear factor kappa B; PPARγ, peroxisome proliferator-activated receptor gamma; PSAP, prosaposin; SLUG, snail family transcriptional repressor 2; TAM, tumor-associated macrophages; TFPI2, tissue factor pathway inhibitor 2; TGF-β, transforming growth factor beta; TGFBI, transforming growth factor beta-induced; TIRAP, TIR domain containing adaptor protein; TMPRSS4, transmembrane protease serine 4; uPA, urokinase-type plasminogen activator; VEC, vascular endothelial cells; VEGF, vascular endothelial growth factor; VSMC, vascular smooth muscle cells.

Nuclear TFPI2 influences cancer-related gene expression and intracellular signaling. Several transcription factors, including activator protein 2 alpha (AP-2α), Sp1 transcription factor (Sp1), Sp3 [43], activating transcription factor 2 (ATF2) [44], GATA binding protein 2 (GATA-2) [45], and p53 [46], regulate MMP-2 transcription. TFPI2 translocates to the nucleus via importin-mediated transport, where it interacts with AP-2α to suppress MMP-2 expression [30]. In breast cancer cells, TFPI2 binds AP-2α, preventing its interaction with the MMP-2 promoter, reducing transcriptional activity, mRNA, and protein levels, thereby inhibiting proliferation and invasion [30] (Figure 2(1)). Thus, TFPI2 suppresses tumors by inhibiting both plasmin-mediated MMP-2 activation and AP-2α-dependent transcription.

#### 3.2.2. Peroxisome Proliferator-Activated Receptor Gamma (PPARγ) and Its Regulation by TFPI2

TFPI2 activates PPARγ, a key regulator of glucose and lipid metabolism [24]. Transcription factors such as C/EBP (CCAAT/enhancer-binding protein) and KLF (Krüppel-like factor) enhance PPARγ transcription, while AP-2α [34], FOXO1 (forkhead box protein O1), and GATA2/3 repress it. TFPI2 interacts with AP-2α, preventing its association with the PPARγ promoter, enhancing expression [34] (Figure 2(2)). PPARγ modulates macrophage polarization, shifting from proinflammatory M1 to anti-inflammatory M2, aiding tissue repair [47,48,49]. However, direct evidence of TFPI2 activating PPARγ specifically in cancer cells remains limited. PPARγ activation may have dual roles in cancer [50]. It induces cell cycle arrest and apoptosis in cancers like esophageal [51] and bladder [52] but promotes tumor growth in prostate cancer [53] by enhancing fatty acid synthesis and mitochondrial biogenesis. In the tumor microenvironment, PPARγ fosters immunosuppressive M2 macrophages and increases regulatory T cells, promoting metastasis [49,50,54]. While PPARγ inhibits cancer cell proliferation, its effects on immune modulation may inadvertently support tumor progression.

#### 3.2.3. Nuclear Factor Kappa B (NF-κB) and Its Regulation by TFPI2

NF-κB regulates cell survival, proliferation, and immune responses [55]. In malignancies, its persistent activation drives tumor growth and apoptosis resistance [56]. TFPI2 induces inflammatory mediators like high mobility group box 1 (HMGB1), which interacts with toll like receptor 4 (TLR4) and receptor for advanced glycation end products (RAGE), establishing a feedback loop that amplifies NF-κB activation, promoting inflammation and tumor progression (Figure 2(3)) [55]. Thrombin upregulates TFPI2 via extracellular signal-regulated kinase 1 and 2 (ERK1/2) and c-Jun N-terminal kinase (JNK) phosphorylation, culminating in NF-κB activation [57], suggesting that TFPI2 contributes to inflammation-driven tumorigenesis (Figure 2(4)).

#### 3.2.4. Regulation of TFPI2-Dependent NF-κB Activation by CAP-Gly Domain-Containing Linker Protein 1 (CLIP1)

CLIP1 suppresses TFPI2-induced NF-κB activation [55]. TFPI2 interacts with CLIP1 and TIR domain containing adaptor protein (TIRAP), a key adaptor in TLR4-NF-κB signaling, modulating TIRAP ubiquitination (Figure 2(5)) [55]. TFPI2 stabilizes TIRAP, reducing NF-κB activation and inflammation. In ischemia-reperfusion injury, TFPI2-CLIP1 binding inhibits TIRAP ubiquitination, reducing inflammation [55]. TFPI2’s role in NF-κB activation is context-dependent, influenced by protein interactions. However, these findings were derived from hepatocytes, and evidence pertaining to cancer cells remains scarce.

#### 3.2.5. Regulation of the Mitogen-Activated Protein Kinase (MAPK)/ERK Pathway by TFPI2

TFPI2 suppresses breast cancer cell proliferation and invasion through regulation of ERK signaling [13]. Overexpression of TFPI-2 in breast cancer cell lines (e.g., MCF7, T47D) led to decreased phosphorylation of ERK1/2 proteins. This reduction in phosphorylation impeded the translocation of activated ERK1/2 into the nucleus, resulting in suppressed cell proliferation [13].

#### 3.2.6. Regulation of Transforming Growth Factor Beta (TGF-β) Signaling by TFPI2

TGF-β regulates cell proliferation, apoptosis, and immune responses [58,59]. TFPI2 enhances TGF-β/Smad signaling by preventing SMURF2 (SMAD-specific E3 ubiquitin protein ligase 2)-SMAD7 (SMAD family member 7) interactions, facilitating TGF-β pathway activation, and promoting endothelial-to-mesenchymal transition and fibrosis, particularly in diabetic nephropathy (Figure 2(6)) [10].

#### 3.2.7. TFPI2 and Integrin-Mediated Focal Adhesion Dynamics

Focal adhesions link the cytoskeleton to the ECM, promoting tumor invasion [60]. TFPI2 knockout enhances cancer proliferation and invasion via integrin α1/β1 clustering [61,62]. Experimental models using ES-2 ovarian clear cell carcinoma [61] and NCI-H460 non-small cell lung carcinoma cells [62] have demonstrated that TFPI2 knockout enhances cancer cell proliferation and invasion by inducing the clustering of integrin α1 or β1. TFPI2 downregulates integrins, suppressing focal adhesion assembly and metastasis [61,62] (Figure 2(7)).

#### 3.2.8. Cytoskeletal Regulation by TFPI2

Actinin 4 (ACTN4) and Myosin 9 (MYH9) regulate cytoskeletal integrity and motility [63,64]. ACTN4 and MYH9 are critical cytoskeletal components that orchestrate a range of cellular functions, including the maintenance of structural integrity, cell motility, and intracellular signal transduction. Functioning as actin filament cross-linking motor proteins, ACTN4 and MYH9 generate contractile forces vital for diverse cellular activities and are indispensable for preserving cytoskeletal architecture [63,64]. Moreover, these proteins have been implicated in the metastatic cascade, contributing to cancer cell dissemination. Overexpressed ACTN4 enhances invasiveness [65], while MYH9 generates contractile forces for migration [66]. TFPI2 interacts with ACTN4 and MYH9, inhibiting breast cancer cell migration and invasion (Figure 2(8)) [13].

#### 3.2.9. TFPI2-Mediated Regulatory Role of Transmembrane Protease Serine 4 (TMPRSS4)

TMPRSS4 promotes cancer invasion by activating pro-urokinase-type plasminogen activator (pro-uPA), degrading ECM, and inducing epithelial–mesenchymal transition (EMT) [67]. TMPRSS4 upregulates integrin α5 and activates focal adhesion kinase (FAK), ERK, Akt, and Rac family small GTPase 1 (Rac1) [68]. In prostate cancer, it induces snail family transcriptional repressor 2 (SLUG) and cyclin D1, promoting invasion and proliferation [69]. TFPI2 downregulates TMPRSS4 in non-small cell lung cancer, inhibiting tumor progression (Figure 2(9)) [69,70].

#### 3.2.10. TFPI2-Mediated Regulatory Role of Prosaposin (PSAP)

PSAP promotes invasion via EMT, immune evasion, and tumor microenvironment interactions [71]. TFPI2 binds PSAP through its second Kunitz domain [72], mitigating its pro-invasive effects. In fibrosarcoma and glioblastoma, TFPI2 suppresses PSAP-induced invasion [72] (Figure 2(10)).

#### 3.2.11. TFPI2-Mediated Suppression of Melanocyte-Induced Transcription Factor (MITF)

TFPI2 suppresses MITF, a key regulator of proliferation and invasion [73] (Figure 2(11)). By binding the MITF promoter, TFPI2 represses its transcription, reinforcing its tumor-suppressive function [73].

These molecules and signaling pathways regulated by TFPI2 are involved in various cellular processes, including cell proliferation, differentiation, apoptosis, migration, cell metabolism, inflammation, and immune response. While each plays distinct roles, they exhibit interconnected functions in certain biological contexts. Multiple factors—including MMPs, PPARγ, NF-κB, TGF-β, integrins, and various proteases—play significant roles in cancer cell proliferation, invasion, and metastasis. For example, TGF-β can modulate the expression of integrins, which are trans-membrane receptors facilitating cell-ECM adhesion [74]. Conversely, integrins can activate la-tent TGF-β, highlighting a reciprocal regulatory mechanism [74]. MMPs can process latent TGF-β, converting it into its active form [75], thereby influencing ECM remodeling and cell behavior. NF-κB and c-Fos are transcription factors that can be activated by various stimuli, including cytokines and stress signals [76]. PPARγ significantly influences cancer cell proliferation, invasion, and metastasis via alterations in EMT markers [77]. They may cooperate in regulating genes associated with inflammation and cell survival. These interactions underscore the complex network of signaling pathways where these factors converge to regulate critical cellular functions.

**Figure 3:**
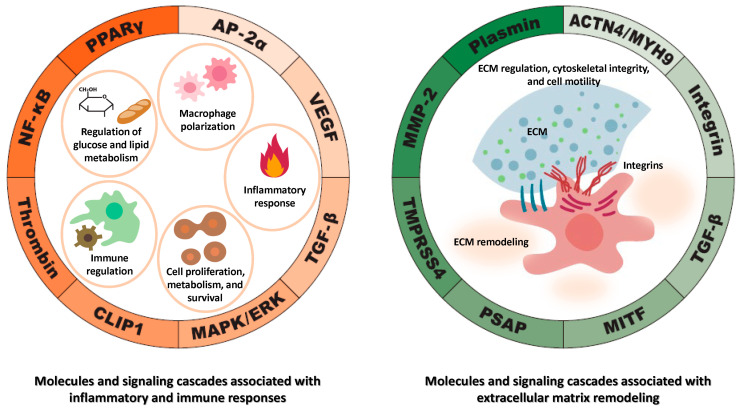
Molecules and signaling pathways regulated by TFPI2. The left pie chart illustrates molecules and signaling pathways modulated by TFPI2 that are implicated in the regulation of glucose and lipid metabolism, macrophage polarization, inflammatory responses, immune modulation, and cellular proliferation and survival. In contrast, the right pie chart depicts molecules and signaling cascades associated with extracellular matrix remodeling, cytoskeletal integrity, and cellular motility.

Figure 3 provides a comprehensive summary of the molecular targets and signaling pathways modulated by TFPI2. TFPI2 modulates a diverse array of molecular processes and signaling pathways, including those involved in glucose and lipid metabolism, macrophage polarization, inflammatory responses, immune regulation, and the regulation of cellular proliferation and survival (Figure 3, left). Additionally, it influences extracellular matrix remodeling, maintenance of cytoskeletal architecture, and cellular motility (Figure 3, right). While each pathway contributes uniquely, they function in a highly interconnected manner within specific biological contexts. These pathways may act synergistically to orchestrate the expression of genes implicated in inflammation, immune responses, cell proliferation, migration, invasion, and survival. Such interactions underscore the intricate network of signaling cascades through which TFPI2 exerts its regulatory influence over critical cellular processes.

### 3.3. Regulation of TFPI2 Expression: Molecular Mechanisms and Signaling Pathways

**Figure 4:**
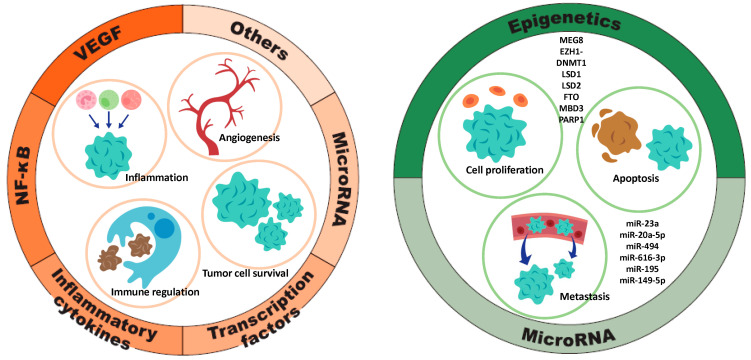
Molecular and signaling pathways governing the regulation of TFPI2 expression. The left pie chart depicts factors that promote the upregulation of TFPI2, while the right pie chart illustrates those that facilitate its downregulation. These genes contribute to cancer invasion and metastasis by modulating inflammatory responses, promoting angiogenesis, regulating immune functions, and influencing tumor cell proliferation, survival, and apoptotic pathways.

The transcriptional and translational regulation of the TFPI2 gene is governed by a diverse array of molecular factors, including DNA methylation, microRNAs (miRNAs), growth factors, cytokines, and tumor-associated signaling pathways, which either promote or suppress its expression [15,16,78]. Upregulation of TFPI2 is mediated by various factors, including VEGF, NF-κB, pro-inflammatory cytokines, transcription factors, and microRNAs (miRNAs), all of which play interrelated roles in the promotion of tumorigenesis through mechanisms involving inflammation, angiogenesis, immune modulation, and tumor cell survival. Conversely, the downregulation of TFPI2 is influenced by epigenetic modifications and specific miRNAs. The interplay between these regulatory elements establishes a complex molecular network that may contribute to oncogenesis by modulating gene expression profiles critical for cellular proliferation, programmed cell death, and metastatic dissemination (Figure 4). While the regulation of TFPI2 in oncogenesis has been extensively reviewed in the recent literature [15,16], this section expands the discussion to include its role in non-tumor pathologies. In the context of malignancies, we specifically highlight ovarian cancer, endometrial cancer, and renal cell carcinoma, where TFPI2 expression is notably upregulated.

#### 3.3.1. Factors Contributing to TFPI2 Upregulation (Figure 4, Left)

##### Signaling Pathway

MAPK signaling pathway: VEGF activates the MAPK/ERK pathway, driving cytoskeletal remodeling and actin reorganization essential for endothelial migration and angiogenesis [79]. VEGF expression is elevated in ovarian and renal carcinomas, particularly in the clear cell subtype, promoting endothelial proliferation and tumor aggressiveness [80]. VEGF also upregulates TFPI2 via the MAPK signaling axis (Figure 2(12)) [29]. In ovarian clear cell carcinoma, high TF expression increases VTE risk via thrombin generation [81]. Thrombin upregulates TFPI2 in hepatic fibroblasts through the MAPK/COX-2 pathway and protease activated receptor 1(PAR-1) signaling (Figure 2(4)) [15,82], a cascade involved in inflammation and prostaglandin synthesis. In THP-1 cells and macrophages, thrombin induces TFPI2 via ERK1/2 and JNK activation [57].

NF-κB pathway: Thrombin triggers TFPI2 expression via NF-κB and activates NF-κB in macrophages through ERK1/2 and JNK pathways (Figure 2(14)) [51]. In hepatocellular carcinoma, TFPI2 inhibits NF-κB-driven MMP expression, suppressing tumor progression (Figure 2(15)) [83]. This bidirectional regulation suggests TFPI2 both responds to and modulates NF-κB, impacting inflammation and cancer. TFPI2 is tightly associated with NF-κB signaling, with their interaction varying by cell type and stimuli. NF-κB is active in ovarian [84], endometrial [85], and renal clear cell carcinomas [86]. In ovarian clear cell carcinoma, it upregulates hepatocyte nuclear factor 1β (HNF-1β) and BCL2 apoptosis regulator (Bcl-2), promoting apoptosis resistance [84,87], and sustains immune evasion and metastasis. In endometrial cancer, NOD-like receptor family CARD do-main-containing 5 (NLRC5) modulates NF-κB to drive progression [88]. In renal clear cell carcinoma, NF-κB enhances angiogenesis by elevating pro-angiogenic markers, while its deletion reduces IL-6 and tumor aggressiveness [86]. Overall, NF-κB activation is common in these cancers, though TFPI2’s role in clear versus non-clear subtypes needs further study.

##### Inflammatory Cytokines

The interaction between TGF-β and TFPI2 is bidirectional, influencing gene expression, signaling, and migration. TFPI2 enhances TGF-β expression, suggesting a positive feedback loop where each upregulates the other [89] (Figure 2(6)). TGF-β2 increases TFPI2 promoter activity and protein levels in renal endothelial cells, supporting its role in fibrosis [10]. Transforming growth factor beta-induced (TGFBI), an ECM protein induced by TGF-β, regulates adhesion, migration, and tissue repair [10] (Figure 2(16)). MYCN pro-to-oncogene (MYCN) suppresses TFPI2 and promotes tumor invasiveness, while TGFBI counteracts this by restoring TFPI2 expression in neuroblastoma [90]. Inflammatory stimuli like TNF-α, endotoxin, phorbol 12-myristate 13-acetate (PMA), and IL-1β elevate TFPI2 expression in normal and malignant cells, including endothelial and glioblastoma cells (Figure 2(17)) [91,92]. These cytokines influence vascular remodeling and angiogenesis [13] and can restore TFPI2 expression, reducing neuroblastoma proliferation and invasion [90]. NF-κB and cytokines are strongly interconnected in inflammation. NF-κB promotes the expression of cytokines, and cytokines can activate NF-κB, forming a loop that drives the inflammatory response [55].

##### Transcription Factors

Activator protein 1 (AP-1), a transcription factor composed of JUN, FOS, and ATF subunits, regulates genes involved in differentiation, proliferation, and apoptosis in response to various stimuli [93]. It promotes TFPI2 expression by binding its promoter (Figure 2(17)) [94]. AP-1 also upregulates TFPI2 during ovulation [94], and its inhibition in cerebral ischemia/reperfusion injury restores blood-brain barrier integrity, indicating AP-1–mediated TFPI2 elevation [95]. p14ARF (CDKN2A) induction enhances c-Jun binding to the TFPI2 promoter, increasing transcription [96]. Furthermore, LIM homeobox domain 6 (LHX6) suppresses Wnt/β-catenin signaling and upregulates TFPI2 through transcription factors like paired-like homeodomain transcription factor 2 (PITX2), contributing to tumor suppression and reduced cancer cell growth [97]. In ovarian cancer, PITX2 overexpression may drive proliferation via genes like cyclin D [98]. Silencing LHX6 in pancreatic cancer enhances proliferation, likely through TFPI2 regulation [97].

##### miRNA/lncRNA

Certain miRNAs like miR-377 and miR-494 and lncRNAs like TFPI2AS1 and AC003092.1 regulate TFPI2 expression [16,78]. miR-377 enhances TFPI2 by downregulating DNA methyltransferase 1 (DNMT1), promoting apoptosis in pancreatic cancer [99]. miR-494 increases TFPI2 mRNA in MCF7 breast cancer cells [78] and suppresses ovarian cancer progression [78,100]. It acts as a tumor suppressor in breast and pancreatic cancers [78,101] but as an oncogene in colorectal cancer [102]. LncRNA TFPI2AS1 upregulates TFPI2, inhibiting G1/S transition and suppressing non-small cell lung cancer growth [78,103]. AC003092.1 boosts TFPI2 through miR-195 inhibition, reducing glioma and gallbladder cancer proliferation [16]. Although miRNA and lncRNA regulation of TFPI2 in ovarian, endometrial, and renal clear cell carcinomas remains underexplored, their expression influences the biology of clear cell carcinomas and their potential as biomarkers [104,105,106,107,108].

##### Others

Estradiol induces TFPI2 expression via estrogen receptor α (ERα) activation in MCF7 breast cancer cells [109]. Human chorionic gonadotropin (hCG) upregulates TFPI2 in granulosa and thecal cells through PPARγ, regulating ovulation and ECM remodeling [28,110]. In HCC, all-trans retinoic acid (ATRA) enhances TFPI2 via retinoic acid receptor alpha (RARα), modulated by MAF bZIP transcription factor B (MAFB, activator) and MAFF (repressor), influencing tumor invasion [111,112]. High calcium in human renal interstitial fibroblasts increases TFPI2, Dachsous Cadherin-Related 1 (DCHS1), and alkaline phosphatase (ALP) activity while reducing ectonucleotide pyrophosphatase/phosphodiesterase 1 (ENPP1), promoting calcification [113]. Curcumin inhibits pancreatic cancer cell invasion by upregulating TFPI2 and suppressing ERK- and JNK-driven EMT [114].

Collectively, VEGF, NF-κB, cytokines, AP-1, miRNAs, and lncRNAs are key regulators in interconnected signaling networks governing cellular functions. NF-κB drives pro-inflammatory cytokine and VEGF expression, promoting tumor angiogenesis [115] (Figure 4, left). Cytokines activate AP-1, which, alongside NF-κB, controls genes linked to proliferation and inflammation. miRNAs and lncRNAs modulate NF-κB signaling and vice versa, forming regulatory loops [116]. lncRNAs also sequester miRNAs, influencing gene expression in inflammation and cancer [117]. Together, these epigenetic factors orchestrate critical processes like immune response, angiogenesis, and tumor progression. The epigenetic regulation of TFPI2 is anticipated to be an increasingly prominent focus in future cancer research.

#### 3.3.2. Factors That Downregulate TFPI2 Expression (Figure 4, Right)

##### miRNA

In pancreatic cancer, miR-23a and miR-20a-5p target TFPI2, enhancing proliferation, migration, and invasion [118,119]. miR-23a inhibition protects against myocardial ischemia/reperfusion injury by upregulating TFPI2 following luteolin (a natural flavonoid) treatment [120]. In breast cancer, miR-494 indirectly downregulates TFPI2 by modulating E74-like factor 1 (ELF-1) and aryl hydrocarbon receptor (AHR), promoting tumor growth [78]. Similarly, miR-616-3p suppresses TFPI2 in prostate cancer and preeclampsia, affecting proliferation, migration, and EMT [38,121]. miR-195 also inhibits TFPI2 in glioblastoma, gallbladder cancer, and preeclampsia, where it enhances trophoblast growth via human umbilical cord-derived mesenchymal stem cells vesicles [16,122,123]. miR-149-5p, through the circ_0015382/miR-149-5p/TFPI2 axis, promotes similar effects in trophoblastic cells [124]. These miRNAs modulate TFPI2 to influence proliferation, invasion, and metastasis across diverse contexts. miRNAs suppressing TFPI2 in tumors have been reviewed [15], but their roles in non-tumor tissues are also significant.

##### Epigenetic Regulation of TFPI2 Expression

The lncRNA maternally expressed gene 8 (MEG8) represses TFPI2 by promoting H3K27me3 trimethylation at its promoter [125]. Silencing MEG8 reduces this mark, increasing TFPI2 and inhibiting angiogenesis in endothelial cells [125]. In glioblastoma, lncRNA AGAP2-AS1 recruits lysine-specific histone demethylase 1 (LSD1) and enhancer of zeste homolog 2 (EZH2) to the TFPI2 promoter, leading to H3K27me3 and DNMT1-mediated methylation, silencing TFPI2 and promoting tumor progression [126,127]. This axis also suppresses TFPI2 in periosteum-derived stem cells, aiding fracture repair [128]. Hyperglycemia upregulates DNMT1, downregulating TFPI2 in endothelial cells, though effects vary by tissue and disease context (Figure 2(18)) [10,24,34]. LSD1 and its homolog LSD2 repress TFPI2 by histone demethylation and methylation at the promoter [129]. LSD1 inhibition restores TFPI2 in triple-negative breast cancer [130], and LSD2 knockdown increases TFPI2 in small cell lung cancer [131]. Fat mass and obesity-associated protein (FTO) reduces TFPI2 via N6-methyladenosine (m6A) RNA demethylation, promoting pancreatic cancer and enhancing keratinocyte proliferation and angiogenesis during wound healing [132,133]. Methyl-CpG-binding domain protein 3 (MBD3) silences TFPI2 in HCC by recruiting the nucleosome remodeling and deacetylase (NuRD) complex for histone deacetylation (Figure 2(19)) [134]. While it promotes tumor progression in gastric cancer [135], it acts as a tumor suppressor in pancreatic cancer by modulating TGF-β/Smad signaling [136]. Poly (ADP-ribose) polymerase 1 (PARP1) represses TFPI2 in VSMCs under hyperglycemia by promoting DNA methylation. Inhibition increases TFPI2, limiting cell proliferation and intimal thickening [137].

Taken together, epigenetic mechanisms and microRNAs (miRNAs) are intricately linked in regulating gene expression, often converging on common biological signaling pathways (Figure 4, right). Epigenetic modifications, such as DNA methylation, can influence the expression of miRNAs. For instance, hypermethylation of CpG islands in promoter regions can silence miRNA genes, while hypomethylation may lead to their overexpression [138]. Conversely, certain miRNAs can directly target and downregulate enzymes involved in epigenetic modifications, such as DNA methyltransferases (DNMTs) and histone deacetylases (HDACs) [139]. Alterations in histone modification patterns can either promote or repress the transcription of specific miRNAs, thereby influencing gene expression profiles [138]. These examples underscore the complex crosstalk between epigenetic mechanisms and miRNAs in regulating critical signaling pathways that govern cellular functions and disease states.

### 3.4. Role of TFPI2 in Non-Neoplastic Diseases

**Figure 5:**
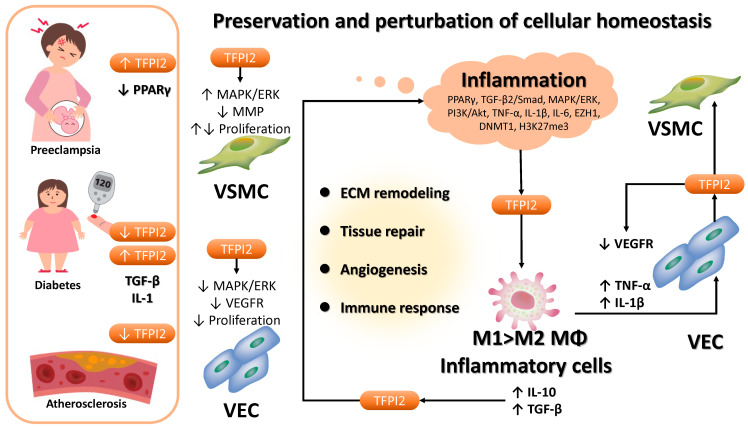
The role of TFPI2 in pregnancy-induced hypertension, diabetes, and atherosclerosis. TFPI2 orchestrates extracellular matrix (ECM) remodeling, tissue regeneration, angiogenesis, and immune modulation. The preservation or disturbance of cellular homeostasis through inflammatory processes may result in TFPI2 dysregulation, thereby contributing to the onset and progression of diabetes, atherosclerosis, and pregnancy-induced hypertension. Superscript arrows represent the upregulation of each factor or the activation of pathological or functional processes, whereas subscript arrows denote downregulation or functional inhibition. DNMT1, DNA methyltransferase 1; ERK, extracellular signal-regulated kinase; EZH1, enhancer of zeste 1 polycomb repressive complex 2 subunit; H3K27me3, tri-methylation at lysine 27 of histone H3; IL-1, interleukin-1; MAPK, mitogen-activated protein kinase; MMP, matrix metalloproteinase; MΦ, macrophage; PI3K, phosphatidylinositol-3-kinase; PPARγ, peroxisome proliferator-activated receptor gamma; TFPI2, tissue factor pathway inhibitor 2; TGF-β, transforming growth factor-beta; TNF-α, tumor necrosis factor-alfa; VEC, vascular endothelial cells; VEGFR, vascular endothelial growth factor receptor; VSMC, vascular smooth muscle cells.

TFPI2 plays a multifaceted role in the pathogenesis of various non-neoplastic conditions, including pregnancy-related disorders such as gestational hypertension [23,38,39,40,41,42], diabetes [10,24,34], and atherosclerosis [35,36,37] (Figure 5). Mechanistically, in vascular smooth muscle cells (VSMCs), TFPI2 facilitates activation of the MAPK/ERK1/2 pathway, enhances cyclin A expression, and promotes mitotic progression [15,140,141,142] (Figure 2(20)). Concurrently, TFPI2 preserves extracellular matrix (ECM) integrity by inhibiting matrix metalloproteinases (MMPs), thereby suppressing aberrant VSMC proliferation and preventing restenosis [7,137,142] (Figure 2(21)). Moreover, TFPI2 exhibits anti-angiogenic properties by attenuating VEGF-induced MAPK/ERK signaling, resulting in reduced proliferation and motility of human umbilical vein endothelial cells (HUVECs) [29] (Figure 2(13)). Clinical studies have demonstrated marked alterations in TFPI2 expression in placental tissues and maternal circulation in cases of gestational hypertension. In such pregnancies, TFPI2 expression is significantly elevated in placental tissues—particularly within syncytiotrophoblasts—compared to normal pregnancies [143], leading to impaired trophoblast proliferation and invasion, and subsequent placental dysfunction [23]. Conversely, maternal plasma TFPI2 levels are notably decreased in women with gestational hypertension relative to normotensive controls [143]. This paradox indicates a complex regulatory dysregulation involving TFPI2 synthesis, release, or clearance in the context of gestational hypertension. In diabetic nephropathy, TFPI2 expression is upregulated in the renal cortex of affected mice [10], where it inhibits VSMC proliferation and migration, thus limiting intimal hyperplasia under diabetic conditions [137]. Additionally, TFPI2 promotes M2 macrophage polarization via PPARγ activation and attenuates fibroblast activation, thereby facilitating myocardial repair post-infarction in diabetic models [24]. Overexpression of TFPI2 enhances plaque stability, induces reparative M2 macrophage polarization, and mitigates hyperglycemia-induced vascular damage [33,36]. In the context of skin aging, TFPI2 downregulation contributes to senescence by impeding cellular proliferation through the PI3K/Akt/CDC6 axis [144]. These findings underscore the protective functions of TFPI2 in atherosclerosis and age-related degeneration [24]. However, contradictory evidence indicates that in diabetic nephropathy, TFPI2 may promote endothelial-to-mesenchymal transition via TGF-β2/Smad signaling, contributing to renal fibrosis and disease progression [10]. These results suggest that TFPI2 influences the migratory and functional properties of various cell types—including endothelial cells, VSMCs, and trophoblasts—in a context-dependent manner. Additionally, TFPI2 expression in non-neoplastic lesions is modulated by epigenetic mechanisms specific to disease state. For instance, EZH1 recruits DNMT1 to the TFPI2 promoter, increasing DNA methylation and H3K27me3, thereby suppressing TFPI2 transcription [128]. This EZH1–DNMT1-mediated repression of TFPI2 enhances osteogenic differentiation in periosteum-derived stem cells and facilitates fracture repair [128]. Another study demonstrated that hyperglycemia induces DNMT1 and downregulates TFPI2 in vascular endothelial cells [34] (Figure 2(18)).

Inflammatory cells, particularly macrophages, play central roles in the pathophysiology of these disorders. This subsection emphasizes the interplay between TFPI2 and macrophages in non-neoplastic inflammatory conditions (Figure 5). In gestational hypertension, the accumulation of inflammatory macrophages disrupts normal placental development [145]. These macrophages secrete pro-inflammatory cytokines such as TNF-α and IL-6, which impede trophoblast invasion and spiral artery remodeling [145]. A distinct shift in placental macrophage polarization toward pro-inflammatory M1 over anti-inflammatory M2 phenotypes is a hallmark of this disorder [145]. Although TFPI2 overexpression in placental tissue is thought to enhance PPARγ levels, PPARγ expression is paradoxically diminished in gestational hypertension [146], contributing to an exacerbated inflammatory milieu [147]. In both type 1 and type 2 diabetes, macrophages exacerbate disease progression by sustaining chronic inflammation [148,149]. These immune cells release pro-inflammatory mediators such as IL-1β, IL-6, and TNF-α, thereby aggravating metabolic dysfunction. Furthermore, macrophages are key contributors to the pathogenesis of atherosclerosis [150], where M1 macrophages predominate over M2, promoting plaque formation and destabilization [24,151,152]. In contrast, M2 macrophages possess anti-inflammatory properties and facilitate plaque stabilization and regression [24,151,152]. The balance between these macrophage subsets critically influences disease trajectory. Pro-inflammatory cytokines such as TNF-α and IL-1β activate endothelial cells, inducing TFPI2 expression [29], which, in turn, suppresses endothelial proliferation by downregulating VEGF receptor expression [29]. Additionally, cytokines like IL-10 and TGF-β secreted by inflammatory cells contribute to immune resolution and tissue repair by modulating TFPI2 activity [153]. Through PPARγ activation, TFPI2 fosters M2 polarization and mitigates vascular injury following myocardial infarction [24,34]. Thus, macrophages, endothelial cells, VSMCs, and other immune components collectively orchestrate the regulation of TFPI2 expression, positioning TFPI2 as a pivotal mediator in inflammation resolution and tissue regeneration.

Taken together, TFPI2 governs ECM remodeling, tissue repair, angiogenesis, and immune modulation. Its dysregulation contributes to the onset and progression of diabetes, atherosclerosis, and gestational hypertension. Macrophages and other immune cells are essential drivers of the underlying inflammatory responses in these diseases, influencing their course through diverse phenotypic states. Analogous to cancer, TFPI2 expression in inflammatory settings is shaped by the surrounding microenvironment and is tightly regulated by immune and inflammatory signals. These findings collectively underscore the indispensable role of TFPI2 in coordinating ECM remodeling, modulating immune responses, and directing cell differentiation in the pathogenesis of non-neoplastic diseases.

### 3.5. Effects of TFPI2 on Cancer Malignancy

#### 3.5.1. Tumor Suppressive Function of TFPI2

TFPI2 downregulation, largely due to promoter hypermethylation, is common in many cancers, including glioma, lung, breast, melanoma, colorectal, pancreatic, liver, gastric, oral, thyroid, cervical, prostate, and bladder cancers [14,15,16,22,154,155,156,157]. In vitro studies show that TFPI2 expression inversely correlates with malignancy in cell lines like glioblastoma, lung cancer, prostate cancer, fibrosarcoma, pancreatic ductal adenocarcinoma, gastric cancer, breast cancer, amelanotic melanoma, and thyroid cancer cells [15]. Overexpression or recombinant TFPI2 suppresses breast cancer cell growth and invasion [30], reinforcing its role as a tumor suppressor [16]. In other words, numerous studies have provided compelling in vitro evidence supporting the role of TFPI2 as a tumor suppressor gene.

#### 3.5.2. Tumor-Promoting Role of TFPI2

Though often considered a tumor suppressor, TFPI2 can promote tumor progression in certain cancers [154,158,159,160]. It is overexpressed in lobular breast carcinoma, hepatocellular carcinoma, and melanoma [15,25,159,160], where it enhances invasion via the TF–factor VIIa complex and supports angiotropism and vasculogenic mimicry (VM), aiding tumor perfusion and correlating with poor prognosis in several cancers [154,160,161]. The aggressiveness of melanoma is intimately associated with the tumor microenvironment (TME), comprising a diverse array of components such as stromal cells, endothelial cells, fibroblasts, blood vessels, immune cells, soluble factors, and the extracellular matrix (ECM) [162]. These elements engage in dynamic, bidirectional interactions with melanoma cells, profoundly impacting tumor growth, progression, and therapeutic responsiveness. Consequently, our review focused on the tumor microenvironment. Changes to the TME, whether through metabolic, mechanical, or inflammatory pathways, can significantly contribute to cancer aggressiveness by creating a supportive environment for tumor growth and spread [163]. Indeed, TFPI2 may promote tumor growth by driving macrophage polarization [24,34,49,50,54]. In glioblastoma, it activates JNK/STAT3 in glioblastoma stem cells (GSCs), enhancing self-renewal and tumor growth [158]. It also recruits and polarizes microglia via the CD51–STAT6 axis, inducing immunosuppression [158,164]. Blocking this pathway restores antitumor immunity in mice [158,164]. Furthermore, TFPI2 may promote M2-type tumor-associated macrophages (TAMs) via PPARγ [24], which support tumor growth through immune evasion, angiogenesis, and ECM remodeling by releasing factors like epidermal growth factor (EGF), hepatocyte growth factor (HGF), VEGF, MMPs, IL-10, and TGF-β [24,153,165,166,167]. In fact, clear cell carcinomas—ovarian, endometrial, and renal—exhibit high TFPI2 and M2 TAM infiltration [168,169,170,171]. These macrophages enhance tumor invasiveness and immunosuppression [168,169,170,171]. Hypoxia-induced exosomes in endometrial cancer upregulate M2 markers via miR-21 [170]. In renal cancer, M2 TAMs are twice as prevalent as in normal tissue, promoting metastasis through midkine (MDK) and chemokines like CXCL13 [171,172,173]. M2 TAMs are also common in TFPI2-overexpressing hepatocellular carcinoma and glioblastoma, where they drive tumor progression via angiogenesis and immune suppression. The tumor-promoting effects of TFPI2 may be particularly pronounced in cancer types that exhibit a strong dependence on the tumor microenvironment.

### 3.6. Impact of TFPI2 on Prognosis

#### 3.6.1. Tissue TFPI2 Levels and Prognostic Significance

Immunohistochemical analysis has identified TFPI2-positive and TFPI2-negative cell populations in malignancies like G3 breast cancer [25], gastric cancer [25], colorectal cancer [25], endometrial cancer [22,25,174], and ovarian cancer [17]. TFPI2 expression inversely correlates with tumor stage and grade, with higher-grade malignancies showing reduced TFPI2 levels [175,176]. Some cancer tissues exhibit heterogeneous TFPI2 staining, with more differentiated tumors showing stronger expression [25]. Metastatic tumors also have higher TFPI2 promoter methylation than localized tumors [15,16,177]. These findings suggest TFPI2 functions as a tumor suppressor, as its expression decreases in advanced stages [25]. Lower TFPI2 levels in tumor cells correlate with poorer survival outcomes in breast, pancreatic, and colorectal cancers [178,179,180]. Data from the Human Protein Atlas (https://www.proteinatlas.org/) (accessed on 30 November 2024) support these findings [181], indicating reduced TFPI2 expression as a negative prognostic marker in many cancers [13,175,182]. TFPI2 has also been detected in tumor-infiltrating macrophages in gastric and renal carcinoma tissues [25].

##### TFPI2 Expression in Ovarian, Endometrial, and Renal Cell Carcinomas

Recent studies have provided more detailed immunohistochemical analyses of TFPI2 expression in ovarian, endometrial, and renal cell carcinoma tissues (Table 2). In a cohort of 142 ovarian cancer cases (77 clear cell carcinomas [CCC] and 65 non-CCCs), TFPI2 expression was observed in 52 cases (36.6%) [17]. Among the 77 CCC cases, 52 (68%) were TFPI2-positive, while none of the 65 non-CCC cases showed expression [17], suggesting TFPI2 as a specific marker for ovarian CCC. However, there was no significant difference in 5-year survival rates between TFPI2-positive and TFPI2-negative ovarian cancer cases [17]. In endometrial cancer, all 13 cases of endometrial clear cell carcinoma showed TFPI2 positivity, while only 11 of 42 non-endometrial clear cell carcinoma cases did [174], indicating TFPI2 specificity for endometrial clear cell carcinoma. However, in a study of 105 type II endometrial cancer cases, TFPI2 was detected in 39 cases (37%), with no significant variation across histological subtypes [22]. Discrepancies may be due to sample composition and variations in antibody concentrations [22]. TFPI2 expression was absent in non-tumor endometrial and myometrial tissues. A study of 54 renal cell carcinoma cases found no significant difference in TFPI2 mRNA expression between renal cell carcinoma and normal renal tissue [19]. Overall, TFPI2 expression is highly specific for ovarian clear cell carcinoma, but its specificity in endometrial and renal cell carcinomas remains unclear. Furthermore, the relationship between TFPI2 expression and prognosis in these cancers requires further investigation.

#### 3.6.2. Serum TFPI2 Levels and Prognostic Significance

Tosoh Corporation has developed an automated immunoassay for quantifying TFPI2 concentrations in blood, using a one-step immunofluorescence technique with two anti-TFPI2 monoclonal antibodies [20]. This assay has been used to assess TFPI2 levels in blood, with a diagnostic threshold of 191 pg/mL showing 64.7% sensitivity, 91.5% specificity, and an AUC of 0.893 for distinguishing ovarian cancer from benign ovarian tumors [20]. Since 2021, TFPI2 testing has been incorporated into Japan’s public health insurance system for ovarian cancer diagnosis [22,73,183]. Elevated serum TFPI2 levels are being explored as biomarkers for VTE and poor prognosis in ovarian cancer [11,12,18]. In ovarian clear cell carcinoma, TFPI2 overexpression may contribute to VTE pathogenesis [102]. Elevated TFPI2 levels have also been linked to poor prognosis in ovarian cancer (Table 2) [18]. In a study of 256 ovarian cancer patients, serum TFPI2 ≥ 255 pg/mL was associated with shorter progression-free survival (PFS) in clear cell carcinoma, and ≥ 201 pg/mL was linked to reduced overall survival (OS) in non-clear cell carcinoma [18]. On the other hand, high levels of miR-20a-5p and miR-616-3p, which downregulate TFPI2, were predictive of VTE occurrence and associated with shorter PFS and OS [119]. In a study of 207 endometrial cancer cases, TFPI2 ≥ 177 pg/mL was associated with advanced age, diabetes, disease stage, and metastasis, with higher levels predicting poorer OS [21]. A study of 328 endometrial cancer patients found that TFPI2 levels increased with disease stage and were linked to poorer five-year survival rates, although no direct correlation with immunohistochemical TFPI2 expression was found [15]. In 42 patients with localized renal cell carcinoma and 12 with metastatic renal cell carcinoma, serum TFPI2 ≥ 170 pg/mL distinguished localized renal cell carcinoma from healthy controls, with levels increasing with tumor grade [19]. No significant correlation was found between serum TFPI2 and tumor size [19]. Overall, serum TFPI2 levels are upregulated in ovarian, endometrial, and renal cancer, with elevated serum levels linked to poor prognosis. However, the relationship between intratumoral TFPI2 expression and clinical outcomes remains unclear.

## 4. Discussion

The inclusion of TFPI2 as an insured cancer diagnostic marker in Japan has spurred heightened research interest. While its biological activity has been investigated in both neoplastic and non-neoplastic contexts, robust multicenter prospective clinical trials have thus far been conducted solely in ovarian cancer [183]. Initially characterized as a tumor suppressor gene, TFPI2 is herein re-examined in light of the paradox that elevated circulating levels are associated with poor prognosis in several malignancies, including ovarian cancer.

**Figure 6:**
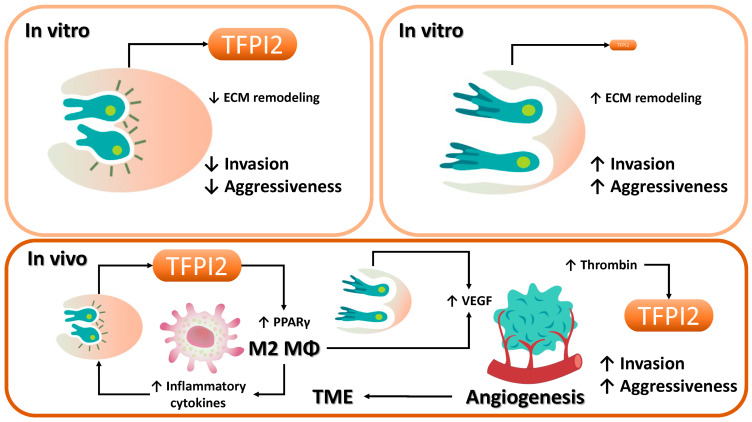
Role of TFPI2 in cancer invasion and metastasis. As illustrated in the upper left panel, TFPI2 overexpression suppresses ECM remodeling and inhibits tumor invasion in vitro. Conversely, the upper right panel shows that TFPI2 downregulation facilitates invasion through MMP-mediated ECM degradation. In vivo, TFPI2 influences not only cancer cells but also diverse components of the TME, including inflammatory and immune cells, endothelial cells, cytokines, ECM constituents, and angiogenic factors, thereby playing a pivotal role in modulating tumor malignancy and metastatic dissemination. Superscript arrows represent the upregulation of each factor or the activation of pathological or functional processes, whereas subscript arrows denote downregulation or functional inhibition.

In vitro studies have demonstrated that TFPI2 functions as a tumor suppressor by inhibiting extracellular matrix (ECM) degradation and restraining tumor cell invasion [14] (Figure 6). However, such in vitro systems often fail to recapitulate the intricate intercellular and structural interactions present in vivo, leading to discrepancies in observed cancer behavior, particularly with respect to invasion and metastasis [184]. Contributing factors include the complexity of the tumor microenvironment (TME), intratumoral heterogeneity, ECM interactions, immune modulation, hypoxic and metabolic gradients, biomechanical stresses (e.g., interstitial fluid pressure and shear stress), and epigenetic modifications [185]. Within the TME, TFPI2 may paradoxically facilitate tumor progression, inducing M2 macrophage polarization and stabilizing VEGF-mediated angiogenesis [153,166]. Notably, in ovarian and endometrial cancers, interactions with M2-polarized macrophages may upregulate TFPI2 expression, thereby enhancing metastatic potential [15]. These findings underscore the context-dependent nature of TFPI2 function within the in vivo tumor milieu [50].

TFPI2 expression is also subject to epigenetic regulation, including DNA methylation and histone modifications, which may serve as clinically relevant biomarkers for cancer diagnosis, prognostication, and individualized therapy (Figure 4, right). The reversibility of epigenetic alterations offers promising avenues for therapeutic intervention. Additionally, the immune landscape of the TME profoundly influences treatment responsiveness [186]. For instance, glioblastoma induces microglial infiltration and immunosuppressive polarization, thereby promoting tumor invasion [158]. In malignancies characterized by substantial immune cell infiltration, immunotherapeutic strategies such as immune checkpoint blockade may prove efficacious [187]. Deciphering the molecular mechanisms underlying TFPI2-driven metastasis may enhance therapeutic precision and contribute to improved clinical outcomes. Beyond oncology, TFPI2 is expressed by inflammatory and endothelial cells and plays roles in non-neoplastic diseases such as diabetes and pregnancy-induced hypertension [188] (Figure 5). It is implicated in modulating inflammation and tissue repair by regulating macrophage and endothelial cell function, suggesting its potential as a novel therapeutic target in inflammatory pathologies.

## 5. Conclusions

TFPI2 exerts diverse biological functions in both tumorigenic and inflammatory settings, with its role intricately modulated by cellular context and microenvironmental cues. Comprehensive understanding of TFPI2 dynamics within the TME is imperative for the development of innovative therapeutic strategies.

## Figures and Tables

**Table 1:** The keyword and search term combinations.

Search Mode	The Keyword and Search Term Combinations
Search term 1	Tissue factor pathway inhibitor 2 OR TFPI2
Search term 2	Ovarian cancer OR Gynecological cancer
Search term 3	Clear cell carcinoma
Search term 4	Tumor suppression OR Suppressor
Search term 5	Tumor promotion OR Promoter
Search term 6	Hyperglycemia
Search term 7	Atherosclerosis
Search	Search term 1
	Search term 1 AND Search term 2
	Search term 1 AND Search term 3
	Search term 1 AND Search term 4
	Search term 1 AND Search term 5
	Search term 1 AND Search term 6
	Search term 1 AND Search term 7

**Table 2:** Prognostic significance of tissue and plasma TFPI2 levels in ovarian, endometrial, and renal cell carcinoma. A blank indicates no data.

Type of Cancer	Ref.	Number of Patients	Tissue	Serum	Prognosis
Ovarian cancer	[17]	142	Fifty-two of seventy-seven ovarian clear cell carcinoma (OCCC) tumors (67.5%) exhibited TFPI2 protein expression in at least one of the nuclear, cytoplasmic, or extracellular matrix compartments, whereas all other histological subtypes (*n* = 65) were TFPI2-negative.		No significant correlation was observed between immunohistochemical findings and five-year survival rates.
			Among 11 OCCC cell lines, four demonstrated TFPI2 expression in the nuclear, cytoplasmic, or extracellular matrix fractions; four were positive exclusively in the extracellular matrix; and three were entirely negative.		
	[18]	256		Serum TFPI2 levels were quantified in 109 serous carcinomas, 66 clear cell carcinomas, and 81 cases of other histological subtypes.	In clear cell carcinoma, patients with serum TFPI2 levels ≥ 255 pg/mL exhibited significantly poorer progression-free survival (PFS) compared to those with lower levels. Among non-clear cell carcinoma cases, patients with serum TFPI2 levels ≥201 pg/mL had significantly reduced overall survival (OS) relative to those with levels below this threshold.
Endometrial cancer	[21]	207			A serum TFPI2 concentration ≥ 177 pg/mL was identified as an adverse prognostic marker.
	[22]	328	Immunohistochemical analysis of 105 endometrial carcinoma cases revealed TFPI2 positivity in 39 cases (37.1%), with no significant variation in expression across histological subtypes.	Serum TFPI2 levels increased in parallel with disease progression and were significantly elevated in high-risk histological variants compared to low-risk types.	A serum TFPI2 level ≥ 191 pg/mL was associated with poor prognosis.
	[174]	55	TFPI2 expression was evaluated via immunohistochemistry in 13 endometrial clear cell carcinoma cases and 42 cases of other histological subtypes. All 13 clear cell carcinoma cases exhibited TFPI2 positivity, whereas 11 cases (26.2%) among the other histological subtypes showed expression.		
Renal cell carcinoma	[19]		No significant difference was detected in TFPI2 mRNA expression between malignant and normal tissues.	Serum TFPI2 levels were assessed in 42 patients with localized disease, 12 with metastatic disease, and 241 healthy controls using a cutoff value of 170 pg/mL. TFPI2 levels were significantly higher in patients with metastatic disease compared to those with localized disease and correlated with tumor grade but not with tumor size.	

## Data Availability

No new data were created.
